# Metabolic resuscitation therapy in critically ill patients with sepsis and septic shock: A pilot prospective randomized controlled trial

**DOI:** 10.1515/med-2023-0637

**Published:** 2023-02-25

**Authors:** Fang Feng, Huyong Yang, Weiwei Yang, Yu Chen

**Affiliations:** Intensive Care Unit of Lanzhou University Second Hospital, No. 82, Cuiying Gate, Chengguanqu, Lanzhou, Gansu 730000, China; Intensive Care Unit of People’s Hospital of Linxia State, Linxia, Gansu 730000, China; Intensive Care Unit of Lanzhou University Second Hospital, Lanzhou, Gansu 730000, China

**Keywords:** metabolic resuscitation, sepsis, septic shock

## Abstract

The main purpose of our research was to further clarify the effectiveness and potential pathophysiological principles of metabolic resuscitation therapy in critically ill patients with sepsis and septic shock. We found that metabolic resuscitation therapy is beneficial for patients with sepsis and septic shock, shortening the length of intensive care unit (ICU) stay, reducing the duration of vasopressor use, and reducing the ICU mortality rate of patients with sepsis and septic shock, but it does not reduce the hospital mortality rate.

## 1 Introduction

Sepsis is a common disease that is easily complicated by a variety of problems. Approximately 1.7 million cases occur annually in the United States, with more than 270,000 deaths [1]. Although intensive medical care has achieved substantial progress, sepsis and septic shock are still the most common causes of death in the intensive care unit. Even if patients with sepsis survive during hospitalization, residual organ dysfunction that requires continued treatment after discharge is common, such as sepsis leading to acute kidney injury followed by chronic renal failure [2].

Ascorbic acid (vitamin C) is a water-soluble vitamin that is essential for many bodily processes. Ascorbic acid, an antioxidant, is an electron donor that directly scavenges free radicals by inhibiting the NADPH oxidase pathway, preventing the formation of new free radicals and assisting with the circulation of other antioxidants [3,4,5]. However, another crucial question is when to administer the first dose of vitamin C. In previous studies, the time to the first dose of vitamin C following admission to hospital was unclear. In our study, as soon as a patient had a suspected infection and the sequential organ failure assessment (qSOFA) score was greater than 2, we administered vitamin C immediately. We are very aggressive in our use of vitamin C. Previous trials [6,7,8] have suggested that the administration of intravenous vitamin C in this setting may have beneficial effects, such as reducing the incidence of organ failure and improving survival.

Recently, Marik et al. [9] published papers in chest describing the application of metabolic resuscitation therapy in patients with sepsis and septic shock. This approach may represent a new approach for treating sepsis and septic shock. Because the trial had a retrospective before-and-after design, we conducted this prospective randomized controlled trial to further clarify the effectiveness, possible pathophysiological principles, and potential applications of metabolic resuscitation therapy in medical patients with sepsis and septic shock.

## 2 Materials and methods

### 2.1 Clinical data

We enrolled medical patients with sepsis and septic shock from September 2019 to March 2020. A tabular form was used to collect the following information: age, sex, use of mechanical ventilation, arterial lactate level at admission and 6 h after admission (GEM3000 blood gas analyser), procalcitonin level at admission and 72 h after admission (using the VIDAS BRAHMS procalcitonin assay, BioMerieux, Inc., Marcy l ‘Etoile, France), use of vasopressin, and acute physiology, age, and chronic health evaluation (APACHE) II score.

### 2.2 Design

The study was a pilot prospective, randomized controlled trial. This trial was approved by the ethics committee of Lanzhou University Second Hospital. Randomization was performed with the use of a centralized computer-generated assignment sequence on the first day in the intensive care unit (ICU). Block randomization was used as a method of random grouping. Complete randomization was applied in our study. First, patients were ranked according to the order of enrolment. A set of random numbers (10) was then assigned to the patients in the same order. Then, the random number column was ranked from smallest to largest, with the first five for the experimental group and the last five for the control group. The intervention treatment was initiated on the first day. The control group received standard care only, and the experimental group received standard care and metabolic resuscitation, including vitamin C (1.5 g in an intravenous infusion q 6 h for 3 days), vitamin B1 (200 mg in an intramuscular injection q 12 h for 3 days), and hydrocortisone (50 mg in an intravenous infusion q 6 h for 7 days). If adequate fluid resuscitation and vasoactive agents failed to stabilize the haemodynamics, steroids were added.

#### 2.2.1 Sample size

According to the previous treatment of patients with sepsis in our hospital, it was estimated that the ICU mortality in the control group was 20%. The power for the primary endpoint was calculated based on a two-sided *t* test with a significance level of 5% using PASS 11 software, with a sample size of 62 subjects treated with metabolic resuscitation therapy and 54 subjects treated with placebo. The trial had more than 80% power to detect a difference between metabolic resuscitation therapy and placebo. If the rate of loss to follow-up was 10%, the sample size of the metabolic resuscitation therapy group was 62/0.9 = 68 subjects and that of the control group was 54/0.9 = 60 subjects.


**Ethics statement:** The Ethics Committee of LanZhou University Second Hospital approved this study, and the number is 2018-043. The Chinese Clinical Trial Registry number is ChiCTR1900026084. All the included patients or their families provided written informed consent (if the patient was unable to sign the form due to the use of sedatives, coma, etc., informed consent was provided by the patient’s immediate family).

### 2.3 Inclusion and exclusion criteria

#### 2.3.1 Inclusion criteria

(1) Consistent with the diagnostic criteria for sepsis 3.0, patients were diagnosed with sepsis when a definite or suspicious infective focus was present, the qSOFA score was greater than or equal to 2 (score standard: systolic pressure ≤100 mm Hg, 1 point; GCS ≤13, 1 point; and respiratory rate greater than 22 breaths per minute, 1 point), and there was evidence of organ failure.
(2) Procalcitonin >2 ng/mL.

#### 2.3.2 Exclusion criteria

(1) Age <18 years;
(2) Pregnant women;
(3) End-stage liver disease; and
(4) G6PD deficiency.

### 2.4 Methods

According to the previous treatment of patients with sepsis in our hospital, it is estimated that the ICU mortality in the control group is 20%. We calculated that we would need to enrol 140 patients for the trial to have 80% power to show ICU mortality at a two-sided alpha level of 5%. Patients with sepsis and septic shock who were admitted to the ICU from September 2019 to March 2020 were prospectively enrolled. According to the computerized random sequence table, the patients were randomly divided into the experimental group and the control group (the random sequence was placed in a sealed, numbered envelope with no light transmission). When patients meeting the criteria were included, the envelopes were selected, and the patients were randomly grouped. All patients with sepsis were included in the research cluster of initial therapy (measurement of arterial lactate level, adequate fluid resuscitation, empiric broad-spectrum antibiotics, vasoactive agents [noradrenaline was preferred; when the dosage of norepinephrine >20 μg/min, it was combined with 0.03 U/min vasopressin] and maintenance of mean arterial pressure [MAP] >65 mm Hg) (see Appendix 1).

### 2.5 Outcomes

The primary outcomes were ICU mortality rate and hospital mortality rate.

The secondary outcomes included the duration of vasoactive drugs (time from the initial use of the vasoactive drugs to the time of withdrawal; the preferred injection of norepinephrine was used to maintain MAP >65 mm Hg, and when the dosage of norepinephrine was greater than 20 μg/min, then 0.03 U/min vasopressin was added); 72 h procalcitonin clearance rate (initial procalcitonin minus procalcitonin at 72 h, divided by the initial procalcitonin and multiplied by 100) at admission; 6 h arterial lactate clearance rate (initial arterial lactate level minus the arterial lactate level at 6 h after admission, divided by the initial arterial lactate value and multiplied by 100); and ICU length of stay (ICU LOS).

### 2.6 Statistical analysis

SPSS 21.0 software (SPSS Inc., Chicago, IL, USA) was used for statistical analysis of the data. If the measured data displayed a normal distribution, the means ± standard deviations (*x* ± *s*) were reported. The *t* test was used to compare the data between the two groups, and the *q*–*q* normal probability graph was used for the normality test. If the measured data did not display a normal distribution, the median (*M*) and interquartile ranges (QL, Qu) were used to present the measured data. Count data were reported as *n* (%). Pearson’s test was used to compare the dichotomous data between the two groups, and the Mann–Whitney *U* test was used for the ordered classification of multiple groups. All tests were two-sided, and *P* < 0.05 was defined as a statistically significant difference.

## 3 Results

### 3.1 Study population

**Figure 1:**
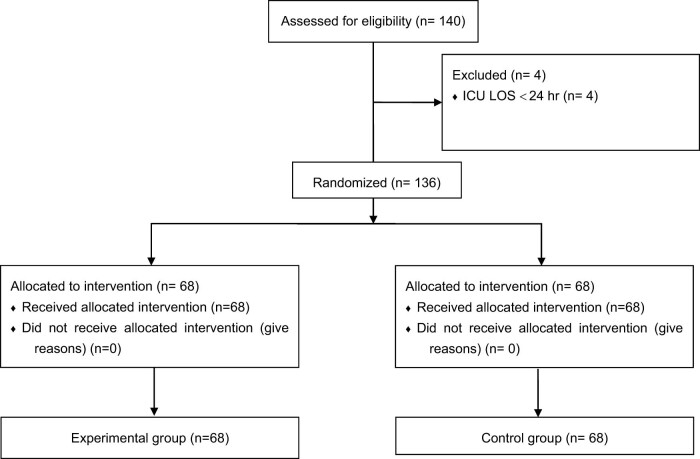
Flow diagram.

According to the inclusion and exclusion criteria, 140 patients were enrolled; among them, the length of the ICU stay of four patients was less than 24 h. There were 17 patients admitted from the wards (10 patients had primary infections that worsened and 7 patients had hospital-acquired secondary infections), and the others were from the emergency department. All patients were medical patients. Then, 136 patients with sepsis and septic shock were included in the study and randomly divided into two groups: 68 in the experimental group and 68 in the control group (Figure 1). Ninety-six percent of patients in the experimental group and 94% in the control group completed standard care. In addition, 100% of patients in the experimental group and 99% in the control group received adequate initial antibiotic treatment (the type of antibiotics was chosen according to the location of infection and the infection indicators). The baseline data collected from the two groups were comparable. Participants in the study were 60% male and 40% female. The mean SOFA score was 8.7 ± 1.3, and the APACHE II score was 11.6 ± 3.2. renal replacement therapy (RRT) for acute kidney injury (AKI) was performed throughout the ICU course. The fluid balance at 6 h was 2,103 ± 140 and 2,009 ± 99 mL in the experimental group and the control group, respectively (*P* = 0.875). The serum creatinine (SCr) at discharge was 75.2 ± 1.7 and 76.1 ± 1.9 µmol/L in the experimental group and the control group, respectively (*P* = 0.349) (Table 1). Among the 136 patients with sepsis and septic shock included in this study, pneumonia was the main cause, as it occurred in 33 (49%) and 41 (60%) patients in the experimental group and the control group, respectively. Gram-negative bacilli were the main pathogenic bacteria (112/136, 82.4%), and there were two negative cultures in the experimental group and three negative cultures in the control group. We describe the bacterial resistance data in Appendix 2. The mean time to the first dose of vitamin C was 1.7 ± 0.2 h (as soon as a patient had a suspected infection and the qSOFA score was greater than 2). Seventeen patients received hydrocortisone in the control group (100 mg q 12 h).

**Table 1:** Baseline information

	Experimental group (*n* = 68)	Control group (*n* = 68)	*P*-value
Age	57.2 ± 14.4	56.7 ± 15.1	0.84
Sex (male)	40 (59%)	42 (62%)	0.727
Infection source			0.881
Pneumonia	33 (49%)	41 (60%)	
BSI	7 (10%)	9 (13%)	
Urosepsis	6 (9%)	4 (6%)	
GI	8 (12%)	6 (9%)	
Other	14 (20%)	8 (12%)	
Comorbidities			
Diabetes	24 (35%)	27 (40%)	0.997
Heart failure	20 (29%)	15 (22%)	0.091
COPD	19 (28%)	21 (31%)	0.085
Hypertension	5 (8%)	4 (7%)	0.254
Mechanical ventilation	37 (54%)	41 (60%)	0.756
Use of vasoactive drugs	32 (47%)	29 (42%)	0.157
Arterial lactate level (mmol/L)	3.8 ± 0.99	3.8 ± 2.4	0.943
Procalcitonin level (ng/mL)	35 (28–100)	31 (19–47)	0.069
CRP (mg/L)	79.4	77.6	0.09
Day 1 SOFA score	9.1 ± 3.2	8.9 ± 2.6	0.274
SOFA score at 72 h	6.9 ± 1.1	7.2 ± 1.4	0.331
Change in SOFA score at 72 h	−3 (−5 to −1)	−1 (−2 to 0)	0.033
APACHE II score	11.9 ± 5.6	11.5 ± 4.7	0.632
APACHE II predicted mortality	15.3 ± 3.2	18.9 ± 4.4	0.259
Adequacy of initial resuscitation	65 (96%)	63 (94%)	0.096
Fluid balance at 6 h (mL)	2,103 ± 140	2,009 ± 99	0.875
Fluid balance at 24 h (mL)	3,414 ± 112	3,387 ± 102	0.783
Adequacy of antibiotic therapy	68 (100%)	67 (99.6%)	0.399
SCr initial (µmol/L)	79.3 ± 2.9	82.1 ± 3.1	0.227
Pathogenic bacteria			
Gram-negative bacilli	55	57	0.49
Negative cultures	2	3	0.83
SCr at discharge (µmol/L)	75.2 ± 1.7	76.1 ± 1.9	0.349
AKI stage			0.099
Stage I	1	3	
Stage II	2	3	
Stage III	0	0	

BSI: blood stream infection; CRP: C-reactive protein.

### 3.2 Primary outcomes

**Figure 2:**
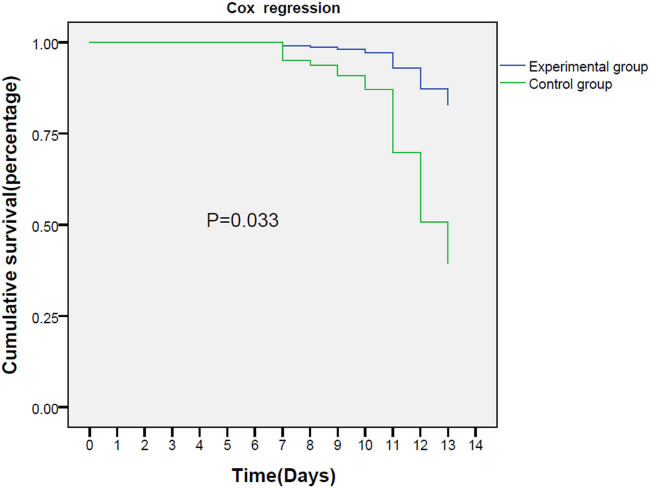
Cox regression.

The ICU mortality rates were 8.8% (6/68) and 15% (15/68) in the experimental group and the control group, respectively (*P* = 0.033). The hospital mortality rates were 16.2% (11/68) and 25% (17/68) in the experimental group and the control group, respectively (*P* = 0.071). The Cox regression is shown in Figure 2.

### 3.3 Secondary outcomes

The ICU length of stay for the experimental group and the control group was 9 (7–12) and 11 (9–14) days, respectively (*P* = 0.002). The duration of vasoactive drug use (h) was 20.8 ± 9.9 and 46.7 ± 12.8, respectively (*P* < 0.001). The 6 h lactate clearance rates were 66.2% (55.5, 76.7) and 30.1% (15.6, 50) in the experimental group and the control group, respectively (*P* < 0.001). The 72 h procalcitonin clearance rates were 70% (57.5, 80.3) and 40.7% (24.8, 52.2), respectively (*P* < 0.001). The full results are shown in Table 2.

**Table 2:** Outcome and treatment variables

	Experimental group (*n* = 68)	Control group (*n* = 68)	*P*-value
ICU LOS (days)	9 (7–12)	11 (9–14)	0.002
Duration of vasoactive drug use (h)	20.8 ± 9.9	46.7 ± 12.8	<0.001
Lactate clearance rate (6 h)	66.2% (55.5, 76.7)	30.1% (15.6, 50)	<0.001
RRT for AKI	1	2	0.075
Procalcitonin clearance rate (72 h)	70% (57.5, 80.3)	40.7% (24.8, 52.2)	<0.001
ICU mortality rate (%)	8.8% (6/68)	15% (15/68)	0.033
Hospital mortality rate (%)	16.2% (11/68)	25% (17/68)	0.071

**Figure 3:**
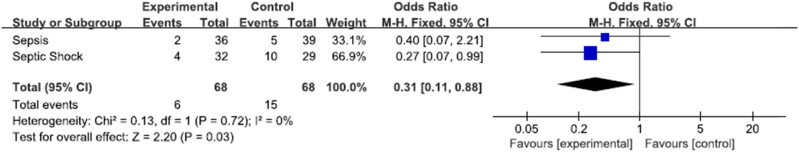
Subgroup analysis of patients with sepsis versus septic shock.

**Figure 4:**
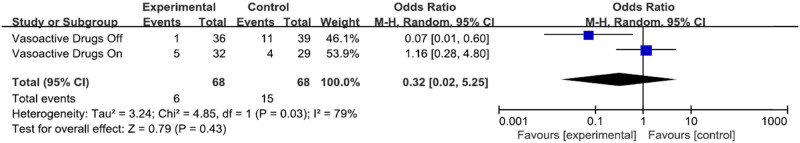
Subgroup analysis of patients who received vasoactive drugs versus those who did not.

We also performed a subgroup analysis of patients with sepsis versus septic shock and patients who received vasoactive drugs versus patients who did not. The results showed that the mortality between sepsis and septic shock was significantly different, with a *P*-value of 0.04; however, no significant difference was observed between patients who received vasoactive drugs and those who did not (Figures 3 and 4).

## 4 Discussion

The fundamental pathogenesis of sepsis is not yet understood and involves many aspects, such as complex systemic inflammatory network effects, gene polymorphisms, immune dysfunction, coagulation dysfunction, tissue damage, and the host’s abnormal response to different infectious pathogens and their toxins. Over the past 30 years, a substantial amount of research and improved clinical processes have increased the speed of recognition and treatment of sepsis. However, the results are still inconsistent. We noticed that the initial time to receive intervention was particularly important. Long MT [10] performed a retrospective cohort study of 208 patients with septic shock and showed that APACHE-adjusted ICU mortality was significantly reduced in patients who received vitamin C, thiamine and steroids in sepsis within 6 h compared with patients who received standard care (odds ratio 0.075 [0.0, 0.59], *P*  < 0.01). Another retrospective study [11] found that delays in the administration of hydrocortisone, vitamin C, and thiamine beyond 12 h after the presentation of sepsis had no influence on patient outcomes, while early therapy was associated with substantial effects.

In our study, the early use of vitamin C in sepsis patients yielded satisfactory results, which further validates the advantage of vitamin C in reversing shock reported in previous studies.

Thiamine (vitamin B1) is a water-soluble vitamin that is a key component of cellular metabolism. Its phosphorylated form, thiamine pyrophosphate, functions as a cofactor of pyruvate dehydrogenase, converting pyruvate to acetyl-CoA and entering the tricarboxylic acid cycle. When the thiamine content is insufficient, pyruvate is not converted to acetyl-CoA, causing impairment in the aerobic chain and a shift to the anaerobic pathway, leading to elevated serum lactate levels [12,13,14]. A retrospective, single-centre, matched cohort study [15] showed that thiamine administration within 24 h of admission in patients presenting with septic shock was associated with improved lactate clearance and a reduction in 28 day mortality compared with matched controls. The research performed by Donnino and colleagues showed that in those with baseline thiamine deficiency, patients in the thiamine group had significantly lower lactate levels at 24 h and a possible decrease in mortality over time [16]. Therefore, the concentration of thiamine should be monitored in patients with sepsis and septic shock in future studies so that thiamine supplementation can be more effective.

Many large-scale randomized trials have evaluated the additional benefits of corticosteroids as part of the treatment for septic shock. According to these studies, corticosteroids generally improve a variety of clinical outcomes of septic shock (e.g., time to shock reversal and number of ventilation-free days) but have different outcomes for mortality [17,18,19]. The routine use of hydrocortisone in patients with septic shock remains a topic of debate [20,21]. However, the biological basis for the inclusion of hydrocortisone in drug combinations is based on the potential synergistic effects of ascorbic acid and hydrocortisone [22]. The absorption of ascorbic acid in cells is mediated by the sodium-vitamin C transporter (SVCT2), whose expression is downregulated in response to inflammation. The use of glucocorticoids has been shown to increase the expression of transporters [23,24]. Previous randomized controlled trials (RCTs) have been published regarding the same intervention in septic patients. Until now, the role of metabolic resuscitation in patients with sepsis and septic shock has remained controversial. Therefore, our trial was designed as a prospective randomized controlled trial to further clarify the role of metabolic resuscitation in patients with sepsis and septic shock. Our study showed that the mortality rate of both groups was lower than that in previous studies (the VITAMINS trial was 19.6%, the HYVCTTSSS trial was 27.5%, and the ORANGES trial was 9%). There may be several reasons. First, the included patients had an APACHE II score of less than 15, so it was possible to conclude that the mortality rate was lower than that in previous studies. Second, the 6 h lactate clearance rate and the 72 h procalcitonin clearance rate were significantly higher than those of the control group, so the timely initiation of antibiotic therapy and fluid resuscitation may also have contributed to the lower mortality rate compared with previous studies. Finally, the prognosis was better than that of conventional RCTs, probably because the intervention was performed early, especially the first dose of vitamin C administration. In the original plan, vitamin B1 was injected intravenously, but because the intravenous route was not mentioned in the specification for vitamin B1 in China, intramuscular injection was used instead.

Another finding in our study is unchanged hospital mortality with improved ICU mortality; this is not the first publication on vitamin C that has seen this result. A retrospective study [11] showed the same results. The application of HAT may improve early shock symptoms, but some patients eventually die from a variety of complications. In this case, there was an improvement in ICU mortality but no difference in hospital mortality.

## 5 Limitations

Our study still has several limitations. First, it is a single-centre study that included a homogeneous patient population. Second, we did not measure the concentration of vitamin C. Finally, our study was designed solely according to the protocol reported by Marik. Although the results showed that metabolic resuscitation may reduce the mortality associated with sepsis and septic shock in patients, the results should be interpreted with caution, and the mechanism by which metabolic resuscitation therapy treats sepsis requires further study in the future.

## 6 Conclusions

Metabolic resuscitation therapy is beneficial for patients with sepsis and septic shock, shortening the length of ICU stay, reducing the duration of vasopressor use, and reducing the ICU mortality rate, but it does not reduce the hospital mortality rate.

## Appendix

### Appendix 1

Standard care:

1. Measure lactate level. Re-measure if initial lactate is > 2 mmol/L;
2. Obtain blood cultures prior to administration of antibiotics;
3. Administer broad-spectrum antibiotics;(One or more antimicrobial agents are administered intravenously in order to cover all possible pathogens. Once the results of pathogen culture and drug sensitivity tests are obtained or the patient is confirmed to be free of infection, empirical anti-infective therapy should be restricted or discontinued immediately.Third generation cephalosporin is preferred, and then, according to the infection indicators, e.g.: WBC, PCT and so on. If necessary, we change appropriate antibiotic according to clinical pharmacist's suggestion.)
4. Rapidly administer 30 mL/kg crystalloid for hypotension or lactate ≥4 mmol/L;
5. Apply vasopressors if patient is hypotensive during or after fluid resuscitation to maintainMAP ≥65 mm Hg.

### Appendix 2

A total of 112 cases of 136 patients were cultured with Gram-negative bacilli, detailed information is shown in the table below.

**Table:**

*Escherichia coli*	74	14 (ESBL)
*Pseudomonas aeruginosa*	21	
*Klebsiella pneumoniae*	10	2 (ESBL)
*Acinetobacter baumannii*	7	2 (XDR), 1 (PDR)

The initial antibiotic therapy was guided by clinical pharmacists.
